# Evaluation of Polygenic Scores and CT Imaging in Risk Factor Modification in Patients With Diabetes: Rationale and Design of the VOLTAIRE Study

**DOI:** 10.1002/clc.70363

**Published:** 2026-06-01

**Authors:** Ruofei Chen, Adam J. Nelson, Sophia Zoungas, Robyn A. Clark, Sean Tan, Esther F. Davis, Melissa C. Southey, Domenic Sacca, Andrew Lin, Giuseppe Di Giovanni, Masashi Fujino, Tayla Micheli, Stephen J. Nicholls

**Affiliations:** ^1^ Monash Victorian Heart Institute Monash University Melbourne Victoria Australia; ^2^ School of Public Health and Preventive Medicine Monash University Melbourne Victoria Australia; ^3^ Caring Future Institute, College of Nursing and Health Sciences Flinders University Adelaide South Australia Australia; ^4^ Department of Clinical Pathology The University of Melbourne Melbourne Victoria Australia; ^5^ Cancer Epidemiology Division Cancer Council Victoria Melbourne Victoria Australia

**Keywords:** cardiovascular disease, computed tomography coronary angiography, diabetes, polygenic risk score, risk factor

## Abstract

**Background:**

The contemporary management of type 2 diabetes (T2D) involves a multifaceted model of care integrating intensive management of lipids, blood pressure, and glucose, along with lifestyle modifications. Despite these efforts, cardiovascular disease (CVD) remains a leading cause of morbidity and mortality among patients with T2D, largely due to suboptimal adherence to preventive strategies. The VOLTAIRE trial aims to assess the impact of providing personalized cardiovascular risk information derived from computed tomography coronary angiography (CTCA) and a polygenic risk score (PRS) on cardiovascular risk factor modification.

**Objectives:**

VOLTAIRE evaluates whether integrating CTCA and PRS into risk counseling enhances adherence to lifestyle and pharmacological interventions, ultimately improving cardiovascular outcomes among patients with T2D.

**Methods:**

VOLTAIRE is a prospective three‐arm, parallel‐group, randomized controlled trial enrolling participants aged 40 years or older with T2D and no established atherosclerotic CVD. Participants are randomized 1:1:1 to receive: (1) risk factor counseling plus CTCA result, (2) risk factor counseling plus PRS result, or (3) standard risk factor counseling (control). Nurse‐led motivational interviewing is used for risk counseling. The primary outcome is change in non‐calcified plaque volume measured by serial CTCA at 12 months. Secondary outcomes include low‐density lipoprotein cholesterol levels, adherence to medication, patient engagement, CVD knowledge improvements, and psychological outcomes over 12 months. Enrollment began in August 2023.

**Discussion:**

VOLTAIRE seeks to determine if coupling nurse‐led risk factor counseling with personalized CTCA or PRS information improves cardiovascular outcomes, adherence, and participant engagement in T2D management.

## Graphical abstract

Three‐arm randomized trial assessing whether computed tomography coronary angiography or polygenic risk score disclosure, combined with nurse‐led counseling, improves cardiovascular risk factor modification in people with type 2 diabetes.

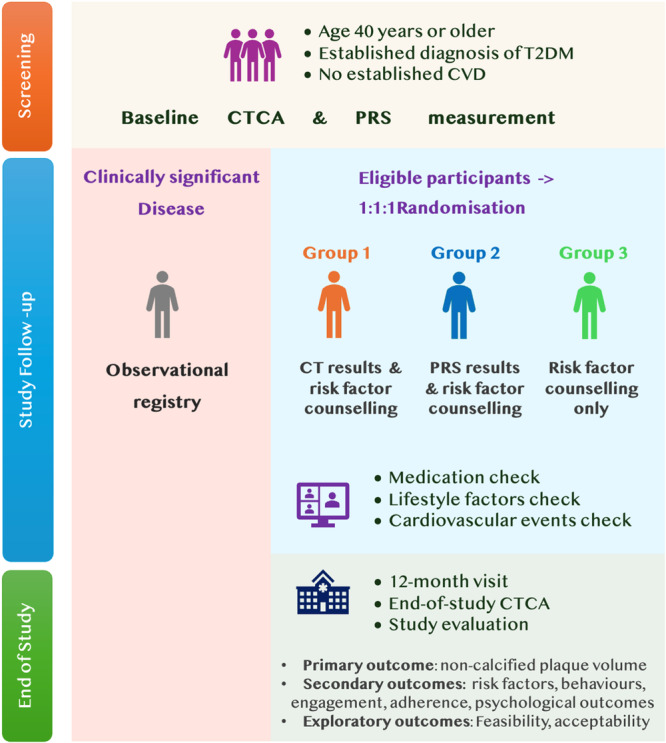

**Abbreviations**
CAC: coronary artery calcium
CAD: coronary artery disease
CTCA: computed tomography coronary angiography
CVD: cardiovascular disease
PRS: polygenic risk score
T2D: type 2 diabetes
VOLTAIRE: EValuation Of poLygenic scores and CT imAging In Risk factor modification in patients with diabetes

## 1 Introduction

Cardiovascular disease (CVD) remains a leading cause of morbidity and mortality globally, imposing a significant burden on healthcare systems and economies [1, 2]. The situation is even more complex in patients with type 2 diabetes (T2D), which is an independent risk factor for all forms of CVD [3]. Individuals with T2D are up to 3 times more likely to experience a cardiovascular event compared to those without diabetes, associated with a significant reduction in life expectancy of up to 5 years [4, 5].

Given the significant clinical impact of CVD on patients with T2D, integrated management of both conditions has become increasingly important. Multifactorial approaches targeting hyperglycemia, hypertension, and dyslipidaemia, combined with lifestyle changes, are essential for preventing CVD in this high‐risk population [6, 7]. Effective interventions must include medical management alongside education, counseling, and behavioral support to ensure sustained risk factor control [8]. However, optimal implementation of guideline‐based prevention in T2D remains a global challenge [7].

In recent years, addressing these challenges has led to increasing interest in using advanced tools, such as cardiac computed tomography (CT) imaging and polygenic risk scores (PRS), to improve engagement and enhance behavioral modification. Cardiac CT imaging directly visualizes coronary artery atherosclerosis, guiding risk factor modification and potentially motivating patients by showing their own coronary disease [9, 10]. This visual feedback has been associated with improved medication adherence and commitment to recommendations [11, 12, 13].

Genomic advancements have also highlighted the significant genetic predisposition for coronary artery disease (CAD), with heritability estimates at 40%–60% [14]. Genome‐wide association studies have identified numerous single nucleotide variants contributing to cardiometabolic diseases, enabling the development of PRS [15, 16]. PRS offers a comprehensive estimate of genetic risk, supporting personalized interventions and encouraging healthier behaviors, as shown in some studies [16, 17, 18]. Despite the potential of these tools, existing studies and systematic reviews report mixed evidence on the ability of CT imaging and PRS to produce sustained risk factor modification [19, 20, 21, 22]. Their optimal role in routine care remains unclear.

The EValuation Of poLygenic scores and CT imAging In Risk factor modification in patients with diabetes (VOLTAIRE) trial seeks to address this gap by evaluating whether integrating personalized cardiovascular risk information from CT coronary angiography (CTCA) and a PRS into nurse‐led risk factor counseling for patients with T2D can enhance adherence to lifestyle and pharmacological interventions. By incorporating visual feedback from CTCA and personalized PRS reports, this trial aims to empower patients to take an active role in managing their cardiovascular health, potentially leading to meaningful improvements in risk factor modification.

## 2 Methods

### 2.1 Study Design

**Figure 1:**
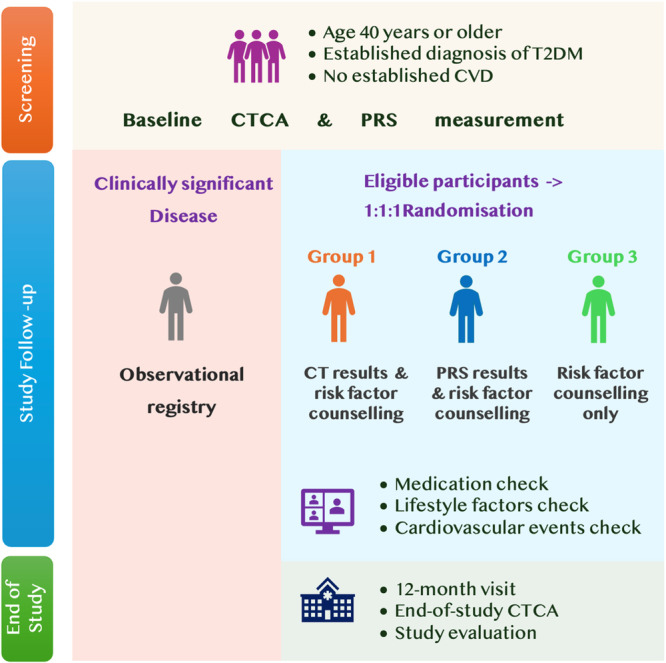
VOLTAIRE trial design. Participants aged ≥ 40 years with T2D and no established CVD undergo baseline assessment including CTCA and PRS measurement. Individuals with clinically significant coronary disease are enrolled in an observational registry. Eligible participants are randomized 1:1:1 to one of three groups: (1) CTCA results with nurse‐led risk factor counseling, (2) PRS results with counseling, or (3) counseling only. Follow‐up includes medication review, lifestyle and cardiovascular event monitoring, and a 12‐month end‐of‐study CTCA and evaluation. Abbreviations: CTCA, Computed tomography coronary angiography; CVD, Cardiovascular disease; PRS, Polygenic risk score; T2DM, Type 2 diabetes mellitus.

The VOLTAIRE trial is a prospective, three‐arm, parallel‐group, randomized controlled trial. Participants are randomized (1:1:1) to: (1) risk factor counseling plus CTCA plaque burden, (2) risk factor counseling plus PRS information, or (3) standard risk factor counseling (control). The study is being conducted at a single site in a metropolitan hospital in Melbourne, Australia, with follow‐up assessments at 6 and 12 months. Recruitment targets individuals with T2D via diabetes outpatient clinics, general practice networks, and community advertisements (flyers, social media) around the hospital and co‐located university campus. This strategy enables a diverse patient sample and supports generalizability. While hospital‐based recruitment may bias toward patients engaged with healthcare, inclusion of community participants and quota sampling for gender balance mitigate this risk. An overview of the trial is presented in Figure 1.

### 2.2 Participants

Inclusion criteria were age ≥ 40 years, T2D managed with lifestyle modification or glucose‐lowering medication, and no history of clinically manifest CVD. Participants required acceptable imaging quality as determined by the Victorian Heart Institute Atherosclerosis Imaging Core Lab (VHI‐AICL).

Exclusion criteria included inability to provide written informed consent, unwillingness to undergo serial evaluation, or clinically manifest CVD. Participants with significant coronary disease on baseline CTCA (≥ 50% stenosis in the left main or ≥ 70% in any epicardial artery) were excluded for safety, as masking would not be possible. Individuals unable to complete study procedures, in the investigator's opinion, were also excluded (Table 1).

**Table 1:** VOLTAIRE eligibility criteria.

Inclusion criteria	Exclusion criteria
Age 40 years or older, Established diagnosis of type 2 diabetes, No history of clinically manifest CVD, Having acceptable imaging quality as deemed by the VHI‐AICL	Unable to provide written informed consent Unwilling to be followed for serial evaluation Clinically manifest CVD Clinically significant coronary disease on CT coronary angiogram (≥ 50% in the left main coronary artery or ≥ 70% in any epicardial coronary artery) Unable to participate in the study or complete protocol required assessments in the opinion of the Investigator

Abbreviations: CTCA, computed tomography coronary angiography; CVD, cardiovascular disease; VHI‐AICL, Victorian Heart Institute Atherosclerosis Imaging Core Lab.

Recruitment ran from August 2023 to June 2025, enrolling 147 participants, with 79 randomized: 26 to the CTCA group, 27 to the PRS group, and 26 to the control group.

### 2.3 Interventions

#### 2.3.1 Conceptual Framework

**Figure 2:**
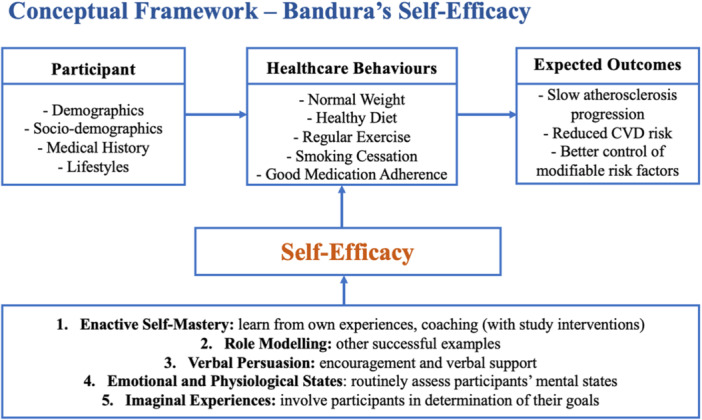
Conceptual framework–Bandura's self‐efficacy. The framework illustrates how participant characteristics influence healthcare behaviors and expected outcomes through self‐efficacy. Self‐efficacy is supported by five sources: enactive self‐mastery, role modeling, verbal persuasion, emotional and physiological states, and imaginal experiences. Improved self‐efficacy promotes behavioral change and cardiovascular risk reduction. Abbreviation: CVD, Cardiovascular disease.

The conceptual framework for the VOLTAIRE trial is based on Bandura's Self‐Efficacy Theory (Figure 2). Self‐efficacy refers to an individual's belief in their ability to perform behaviors that lead to specific outcomes [23, 24, 25]. This theory posits that higher self‐efficacy enhances motivation, persistence, and resilience, thereby improving behavioral change and

performance. In the context of cardiovascular risk management, fostering self‐efficacy can empower participants to engage more effectively in lifestyle changes and adhere to recommended treatments, ultimately improving health outcomes.

#### 2.3.2 CT Coronary Angiography (CTCA)

Participants undergo CTCA at baseline, with a repeat scan at 12 months for those randomized. CTCA is performed using multi‐detector row scanners (Aquilion One Prism or Insight; Canon Medical Systems Corp., Otawara, Japan). Beta‐blockers and sublingual nitroglycerine are administered prior to achieve a heart rate of <65 beats/min and coronary vasodilation. Iodinated contrast (60–90 mL, 350 mg iodine per mL [Omnipaque; GE Healthcare, Chicago, IL, USA]) is injected at 5 mL/s. Acquisition is automatically triggered at 300 Hounsfield units in the arterial phase using bolus tracking in the descending aorta. Scan parameters include detector collimation 280–320 × 0.5 mm, tube current 100–800 mA (body mass index [BMI]‐dependent), and tube voltage 120 kV (BMI ≥ 25 kg/m^2^) or 100 kV (BMI < 25 kg/m^2^). Images are reconstructed with a 512 × 512 matrix, 0.5 mm slice thickness, sharp convolution kernel, and deep learning‐based reconstruction (Advanced intelligent Clear‐IQ Engine [AiCE], Canon Medical Systems, Otawara, Japan). All CTCA scans are clinically co‐reported by an experienced radiologist and cardiologist according to Society of Cardiovascular Computed Tomography guidelines [26]. If no obstructive CAD (≥70% stenosis in a major epicardial coronary arteriy or ≥50 stenosis in the left main) is detected, results remain blinded until trial completion. Participants with obstructive CAD have reports released, undergo clinical follow‐up, and are enrolled in the observational registry. Incidental non‐cardiac findings are communicated to participants and their general practitioners (GP) for follow‐up.

**Figure 3:**
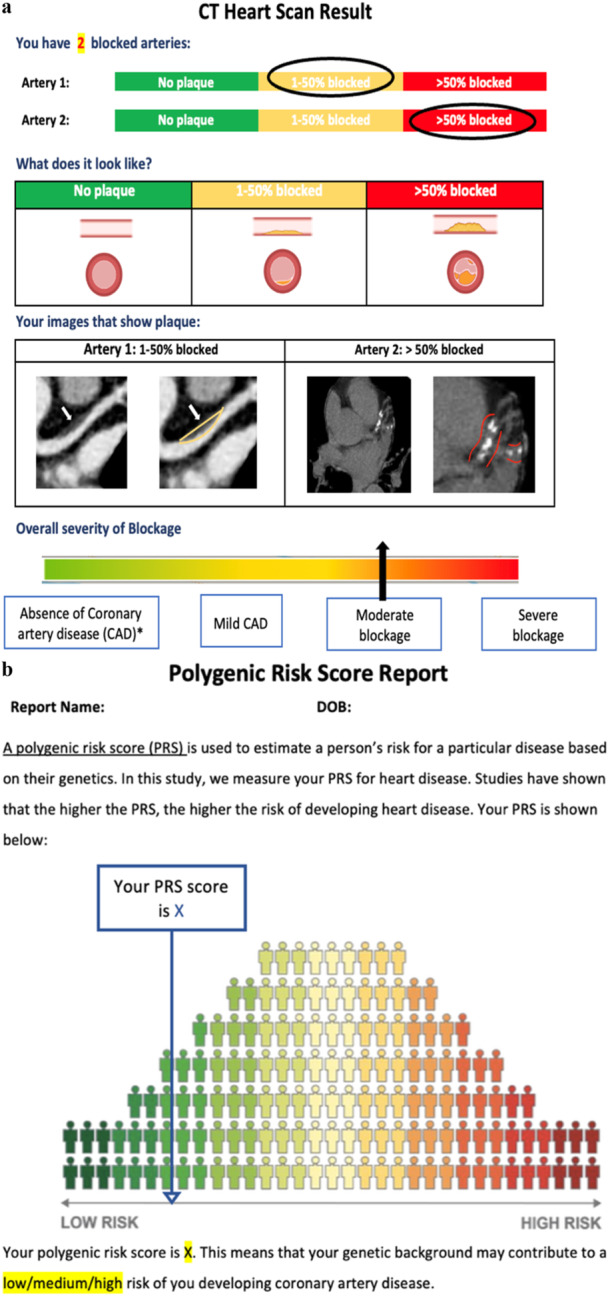
(a) VOLTAIRE trial CT report. This example report visually summarizes plaque presence and severity, and categorizing blockages as no plaque, 1%–50% blocked, or >50% blocked. Illustrations and actual CT images help participants understand the extent of their coronary artery disease. An overall severity scale contextualizes the results, ranging from no disease to severe blockage. (b) VOLTAIRE trial PRS report. This example report illustrates a participant's PRS for heart disease. The visual places the individual's score within a population distribution ranging from low to high genetic risk. Color coding and simple language help communicate risk levels in an accessible and easily understood format.

Participants randomized to receive their CTCA results (Group 1) are given a layperson report co‐designed with Monash Health consumer representatives to ensure clarity and relevance (Figure 3a). The iterative process incorporated consumer feedback to refine content and format. The report summarizes coronary artery stenosis extent in simple terms with visual aids, detailing plaque presence and severity by artery, and categorizing CAD as absent, mild, moderate, or severe. End‐of‐study CT scans use the same equipment and parameters as baseline.

#### 2.3.3 CTCA Quantitative Plaque Analysis

All image analysis will be performed in the VHI‐AICL by trained analysts using licenced artificial intelligence (AI)‐enabled research software (Autoplaque v3.0, Cedars‐Sinai Medical Center, Los Angeles, CA, USA). Image quality will be assessed prior to analysis, with scans excluded if uninterpretable due to artefacts or missing data. The analyst will define proximal and distal plaque limits, after which adaptive scan‐specific Hounsfield unit (HU) thresholds for plaque components will be auto‐generated. Contouring of the vessel wall and lumen will be automatic, with manual adjustment as required. Plaque is defined as all voxels located between the vessel and luminal boundaries. Per‐lesion volumes (mm^3^) will be calculated for total, calcified, non‐calcified, and low‐attenuation plaque (<30HU). The respective plaque burdens (%) will be calculated as: plaque volume ÷ analyzed vessel volume × 100. Plaque volume and burden measurements for the entire coronary tree will be summed on a per‐patient level. Per‐lesion plaque measurements across the coronary tree will be summed at the per‐patient level.

#### 2.3.4 Polygenic Risk Score (PRS)

At baseline, a venous blood sample is collected, processed, and genomic DNA extracted at Biobanking Victoria, Precision Medicine, Monash University. Genotyping is performed using the Confluence array (Illumina, CA, USA), and PRS calculated at The Broad Institute as described previously [27]. The PRS report (Figure 3b), co‐designed with Monash Health consumer representatives, uses simple language and visual aids, such as a bell curve, to contextualize individual risk relative to the general population. Co‐design focused on presenting genomic data clearly while maintaining scientific accuracy to support understanding and informed decision‐making.

#### 2.3.5 Risk Factor Counseling

Risk factor counseling is delivered either in‐person or via telehealth, depending on participants preference, by a trained study nurse. Advice follows recommendations from the International Diabetes Federation [28], American Heart Association Life Essential 8 [29], Diabetes Australia [30], and the Heart Foundation [31]. Motivational interviewing (MI) techniques are used to encourage health‐promoting behaviors and adherence to therapies. MI was selected for its proven ability to foster intrinsic motivation and sustainable behavior change, empowering participants to identify their own reasons for change and enhancing long‐term commitment [32]. Participants in Groups 1 and 2 receive their CTCA or PRS results at initial consultation, with reinforcement at a 6‐month follow‐up. Sessions also involve setting SMART (Specific, Measurable, Achievable, Relevant, Time‐bound) goals to support cardiovascular risk factor modification. Key components are outlined in Table 2.

**Table 2:** Key Components of the intervention.

Components	Description
Health Education	Smoking Cessation: Assess willingness to quit; provide counseling and resources; with consent, refer to Quitline Diet: Review current habits; promote healthy proteins, more vegetables/fruits, low‐GI foods, nutritious snacks, and portion control Physical Activity: Encourage ≥ 150 min/week moderate‐intensity exercise; discuss personalized activity options
Engagement with Allied Health Professionals	Review current engagement; encourage visits to dietitian, exercise physiologist, and diabetes educator for tailored plans, and to podiatrist and optometrist for preventive care
Cardiovascular Risk Assessment	Calculate 5‐year CVD risk using the New Zealand Society for the Study of Diabetes CVD Risk Assessment calculator, which incorporates diabetes duration, glycated hemoglobin, blood pressure, lipid profile, urine albumin‐creatinine ratio, and antihypertensive use

Abbreviation: CVD, cardiovascular disease.

### 2.4 Outcomes

The primary objective is to assess the impact of personalized cardiovascular risk information, derived from CTCA and PRS, on cardiovascular risk factor modification in individuals with T2D (Table 3). The primary outcome is change in non‐calcified plaque volume on CTCA at 12 months. Non‐calcified plaque volume was selected because it reflects a more biologically active component of coronary atherosclerosis and may be more responsive to risk factor modification over relatively short timeframes.

**Table 3:** VOLTAIRE trial objectives and outcomes.

Primary outcome
– Change in non‐calcified plaque volume
Secondary outcomes
– Achievement of an LDL‐C < 1.8 mmol/L – Intensification of lipid‐lowering therapy, defined as any of the following—increase in the intensity of statin prescribed, addition of ezetimibe, PCSK‐9i – Prescription of a guideline‐recommended statin – Reporting of high levels of adherence to their lipid‐lowering therapy – Level of patient activation and engagement in their care during the study period, compared with baseline – Mental health responses (psychological and emotional reactions) after receiving PRS or CTCA results, including personal perceived risk, personal perceived health‐related quality of life, anxiety, depression or other mental health issues – Change in 5‐year CVD risk
Exploratory outcomes
– Incidence of cardiovascular events – Achievement of all three modifiable risk factor goals: LDL‐C < 1.8 mmol/L, systolic blood. pressure ≤ 130 mmHg, and smoking cessation – Change in hsCRP – Change in HbA1c – Changes in additional imaging parameters, including total atheroma volume, calcified plaque volume, percent atheroma volume, PCAT and epicardial fat volume and density – Achievement of an LDL‐C < 1.8 mmol/L at any time during follow up
Implementation outcome
– Appropriateness, feasibility, and acceptability of the CTCA or the PRS intervention from participants' perspectives

Abbreviations: AE, adverse event; CTCA, computed tomography coronary angiography; CVD, cardiovascular disease; HbA1c, glycated hemoglobin A1c; hsCRP, high‐sensitivity C‐reactive protein; LDL‐C, low‐density lipoprotein cholesterol; PCAT, pericoronary adipose tissue; PCSK‐9i, proprotein convertase subtilisin/kexin type 9 inhibitors; PRS, polygenic risk score.

Secondary outcomes include the achievement of Low‐density lipoprotein‐cholesterol (LDL‐C) levels below 1.8 mmol/L at 12 months, intensification of lipid‐lowering therapy, prescription of guideline‐recommended statins, and adherence at 6 and 12 months. Additional measures include changes in patient activation (PAM‐13), psychological health (depression, anxiety, perceived risk, quality of life), and 5‐year CVD risk scores.

Exploratory outcomes include major cardiovascular events (e.g., all‐cause death, myocardial infarction or stroke), achievement of modifiable risk factor targets (LDL‐C < 1.8 mmol/L, systolic BP ≤ 130 mmHg, and smoking cessation), changes in biomarkers (HbA1c, hsCRP), and other imaging parameters (total atheroma volume, calcified plaque volume). Implementation objectives assess the appropriateness, feasibility, and acceptability of the CTCA or PRS intervention from participants' perspectives.

### 2.5 Randomization and Blinding

Participants are randomized (1:1:1) using a computer‐generated random sequence with allocation concealment, conducted by research staff not involved in participant interactions to prevent selection bias. Outcome assessors are blinded to minimize analysis bias. Due to the nature of the interventions, participants and their treating clinicians are not blinded; however, site staff remain blinded to imaging results in non‐CTCA groups and PRS results in non‐PRS groups.

### 2.6 Data Collection

Data collection includes comprehensive assessments at baseline, 6 months, and 12 months, covering socio‐demographic, clinical, behavioral, and psychological measures. Details of all assessments, including timelines and specific parameters, are provided in Table S1.

### 2.7 Statistical Analysis

#### 2.7.1 Sample Size Calculation

The sample size was determined based on the objective to compare progression rates across the three groups. A total of 30 participants per treatment group (total *n* = 90) is required to undergo serial, evaluable imaging. This sample size provides 80% power to detect a difference in the progression of non‐calcified plaque volume between a pair of groups of 8 mm^3^, assuming a standard deviation of 11 mm^3^, a Bonferroni‐adjusted alpha of 0.83%, and that baseline adjustment accounts for one‐third of outcome variability.

#### 2.7.2 Statistical Methods

Analyses will follow a pre‐defined statistical analysis plan on an intention‐to‐treat basis, with two‐sided tests and a significance level of *α* = 0.05. Descriptive statistics will summarize socio‐demographic, clinical, and health profiles. Categorical data will be presented as frequencies and compared using chi‐square tests, reporting odds ratios and 95% confidence intervals. Continuous variables will be presented as either mean with standard deviation or median with interquartile range, with group comparisons via independent *t*‐tests or Mann‐Whitney *U* tests as appropriate.

Primary and secondary endpoints between groups at 12 months will be analyzed using mixed‐effects models, adjusting for baseline values and relevant socio‐demographic confounders, to assess treatment effects over time. Implementation objectives will be evaluated using a mixed methods approach: a 19‐question survey (including two free‐text sections) and focus groups (5–10 participants per intervention group), with qualitative data analyzed thematically.

### 2.8 Ethics and Governance

#### 2.8.1 Ethics Approval

The study complied with the National Statement on Ethical Conduct in Human Research by the Australian National Health and Medical Research Council (National Health and Medical Research Council, 2018) and was approved by Monash University Health Research Ethics Committee (Project number: RES‐22‐0000‐656A).

#### 2.8.2 Data Management

Participant data were stored in an encrypted, secure electronic database (REDCap) hosted by Monash University. with access limited to authorized staff. Data were de‐identified for analysis, and records will be retained for 15 years in accordance with guidelines.

#### 2.8.3 Trial Registration

ClinicalTrials. gov ID: NCT07091162.

## 3 Discussion

The VOLTAIRE trial is one of the first studies to evaluate the impact of personalized cardiovascular risk information, derived from CTCA and PRS, on cardiovascular risk factor modification. While effective communication is known to motivate behavioral changes and improve adherence, the specific impacts of CTCA and PRS in these areas remain underexplored.

Providing patients with vascular plaque information via carotid ultrasound or coronary artery calcium (CAC) imaging can improve risk factor control and medication adherence [11, 33]. However, carotid ultrasound assesses only the carotid arteries, and CAC detects only calcified plaques, potentially missing non‐calcified, vulnerable plaques [9]. By contrast, CTCA visualizes both calcified and non‐calcified plaques, offering a more comprehensive evaluation of CAD [9, 29]. Despite these advantages, evidence on CTCA's ability to drive behavioral change is mixed, likely due to differences in communication, reinforcement, and whether patients received their images [19].

PRS is an emerging tool for identifying genetic predisposition to CAD, with high genetic risk typically conferring a two‐ to threefold higher relative risk than average [27]. Despite this, adherence to a healthy lifestyle can significantly mitigate risk. Khera et al. showed that high‐risk individuals maintaining favorable lifestyles had a 46% lower relative risk of coronary events [27]. Yet, behavioural effects of PRS has been mixed, largely due to differences in communication [21, 34]. A recent systematic review suggests that genetic insights may prompt modest dietary improvements but has little effect on physical activity, medication adherence, or traditional risk factors [35]. To address these challenges, a key recommendation is to contextualize genetic risk by using visual aids [36, 37]. The VOLTAIRE trial adopts this approach, presenting PRS via a bell curve to enhance comprehension and support informed decision‐making.

In addition, VOLTAIRE was designed to address limitations of prior imaging and genetic risk disclosure studies through co‐designed visual reports, structured nurse‐led counseling, personalized recommendations, and reinforcement at follow‐up. CTCA and PRS reports were developed in partnership with hospital consumer representatives, using simple language and visual summaries, to ensure relevance and accessibility. Acceptability and effectiveness will be further evaluated through focus groups.

The VOLTAIRE trial also addresses broader health needs by screening for depression, anxiety, diabetes distress, and sleep apnoea, with referral of severe cases to GPs. This integrated approach recognizes the interplay between physical and psychological health. Recruitment through community GP clinics, diabetes support groups, social media, and local university campuses supported enrollment of a broader and more diverse cohort.

This study should be interpreted considering several limitations. The single‐center design may limit generalizability to other populations and settings, and hospital‐based enrollment may still have preferentially included individuals who were more engaged with healthcare, which could have influenced responsiveness to the interventions. Given the open‐label design, performance bias cannot be excluded, as participants and clinicians were aware of group allocation. The modest sample size was powered for the primary mechanistic endpoint but may have limited statistical power for some secondary and exploratory outcomes. Larger, adequately powered multicentre studies are needed to confirm these findings.

## Author Contributions

**Ruofei Chen (Trophy):** conceptualization, data curation, formal analysis, investigation, methodology, project administration, resources, visualization, writing – original draft, writing – review and editing. **Adam J. Nelson:** conceptualization, methodology, supervision, writing – review and editing. **Sophia Zoungas:** conceptualization, methodology, supervision, writing – review and editing. **Robyn A. Clark:** conceptualization, methodology, supervision, writing – review and editing. **Sean Tan:** project administration, validation, writing – review and editing. **Esther F. Davis:** project administration, validation, writing – review and editing. **Melissa C. Southey:** investigation, resources, writing – review and editing. **Domenic Sacca:** data curation, project administration, resources, writing – review and editing. **Andrew Lin:** formal analysis, resources, writing – review and editing. **Giuseppe Di Giovanni:** project administration, resources, validation, writing – review and editing. **Masashi Fujino:** project administration, resources, validation, writing – review and editing. **Tayla Micheli:** data curation, project administration, resources, writing – review and editing. **Stephen J. Nicholls:** conceptualization, methodology, supervision, writing – review and editing.

## Funding

The authors have nothing to report.

## Conflicts of Interest

The authors declare no conflicts of interest.

## Supporting information

Supporting File

## Data Availability

Data sharing not applicable to this article as no datasets were generated or analyzed during the current study.

## References

[1] Heart Foundation . Key Statistics: Cardiovascular Disease, accessed 6 October, 2024, https://www.heartfoundation.org.au/bundles/for-professionals/key-stats-cardiovascular-disease.

[2] World Health Organization . Cardiovascular Disease, accessed 6 October, 2024, www.who.int/health-topics/cardiovascular-diseases#tab=tab_1.

[3] T. R. Einarson , A. Acs , C. Ludwig , and U. H. Panton , “Prevalence of Cardiovascular Disease in Type 2 Diabetes: A Systematic Literature Review of Scientific Evidence from Across the World in 2007–2017,” Cardiovascular Diabetology 17, no. 1 (2018): 83, 10.1186/s12933-018-0728-6. 29884191 PMC5994068

[4] International Diabetes Federation . Complications, accessed 6 October 2024, https://idf.org/about-diabetes/diabetes-complications/.

[5] O. H. Franco , E. W. Steyerberg , F. B. Hu , J. Mackenbach , and W. Nusselder , “Associations of Diabetes Mellitus With Total Life Expectancy and Life Expectancy With and Without Cardiovascular Disease,” Archives of Internal Medicine 167, no. 11 (2007): 1145–1151, 10.1001/archinte.167.11.1145. 17563022

[6] P. Gæde , P. Vedel , N. Larsen , G. V. H. Jensen , H.‐H. Parving , and O. Pedersen , “Multifactorial Intervention and Cardiovascular Disease in Patients with Type 2 Diabetes,” New England Journal of Medicine 348, no. 5 (2003): 383–393, 10.1056/NEJMoa021778. 12556541

[7] F. Cosentino , P. J. Grant , V. Aboyans , et al., “2019 ESC Guidelines on Diabetes, Pre‐Diabetes, and Cardiovascular Diseases Developed in Collaboration with the EASD: The Task Force for Diabetes, Pre‐Diabetes, and Cardiovascular Diseases of the European Society of Cardiology (ESC) and the European Association for the Study of Diabetes (EASD),” European Heart Journal 41, no. 2 (2020): 255–323, 10.1093/eurheartj/ehz486. 31497854

[8] L. G. Sisti , M. Dajko , P. Campanella , E. Shkurti , W. Ricciardi , and C. de Waure , “The Effect of Multifactorial Lifestyle Interventions on Cardiovascular Risk Factors: A Systematic Review and Meta‐Analysis of Trials Conducted in the General Population and High Risk Groups,” Preventive Medicine 109 (2018): 82–97, 10.1016/j.ypmed.2017.12.027. 29291422

[9] M. N. Meah , M. R. Dweck , and D. E. Newby , “Cardiovascular Imaging to Guide Primary Prevention,” Heart 106, no. 16 (2020): 1267–1275, 10.1136/heartjnl-2019-316217. 32451365

[10] CT Coronary Angiography in Patients with Suspected Angina Due to Coronary Heart Disease (SCOT‐HEART): An Open‐Label, Parallel‐Group, Multicentre Trial,” Lancet 385, no. 9985 (2015): 2383–2391, 10.1016/S0140-6736(15)60291-4. 25788230

[11] P. Venkataraman , Q. Huynh , S. J. Nicholls , T. Stanton , G. F. Watts , and T. H. Marwick , “Impact of a Coronary Artery Calcium‐Guided Statin Treatment Protocol on Cardiovascular Risk at 12 Months: Results from a Pragmatic, Randomised Controlled Trial,” Atherosclerosis 334 (2021): 57–65, 10.1016/j.atherosclerosis.2021.08.002. 34482089

[12] R. E. Mols , J. M. Jensen , N. P. Sand , et al., “Visualization of Coronary Artery Calcification: Influence on Risk Modification,” American Journal of Medicine 128, no. 9 (2015): 1023.e23–1023.e31, 10.1016/j.amjmed.2015.03.033. 25910787

[13] N. K. Kalia , L. Cespedes , G. Youssef , D. Li , and M. J. Budoff , “Motivational Effects of Coronary Artery Calcium Scores on Statin Adherence and Weight Loss,” Coronary Artery Disease 26, no. 3 (2015): 225–230, 10.1097/MCA.0000000000000207. 25514570

[14] R. McPherson and A. Tybjaerg‐Hansen , “Genetics of Coronary Artery Disease,” Circulation Research 118, no. 4 (2016): 564–578, 10.1161/CIRCRESAHA.115.306566. 26892958

[15] N. Mars , J. T. Koskela , P. Ripatti , et al., “Polygenic and Clinical Risk Scores and Their Impact on Age at Onset and Prediction of Cardiometabolic Diseases and Common Cancers,” Nature Medicine 26, no. 4 (2020): 549–557, 10.1038/s41591-020-0800-0. 32273609

[16] A. V. Khera , M. Chaffin , K. G. Aragam , et al., “Genome‐Wide Polygenic Scores for Common Diseases Identify Individuals with Risk Equivalent to Monogenic Mutations,” Nature Genetics 50, no. 9 (2018): 1219–1224, 10.1038/s41588-018-0183-z. 30104762 PMC6128408

[17] E. D. Muse , S. F. Chen , and A. Torkamani , “Monogenic and Polygenic Models of Coronary Artery Disease,” Current Cardiology Reports 23, no. 8 (2021): 107, 10.1007/s11886-021-01540-0. 34196841 PMC8317496

[18] E. Widén , N. Junna , S. Ruotsalainen , et al., “How Communicating Polygenic and Clinical Risk for Atherosclerotic Cardiovascular Disease Impacts Health Behavior: An Observational Follow‐up Study,” Circulation: Genomic and Precision Medicine 15, no. 2 (2022): e003459, 10.1161/CIRCGEN.121.003459. 35130028

[19] R. Chen , A. J. Nelson , S. Tan , R. A. Clark , S. Zoungas , and S. J. Nicholls , “The Effectiveness of Visualising Plaque on Cardiac Computed Tomography in Modifying Risk Factors for Cardiovascular Disease: A Systematic Review,” Journal of Cardiovascular Computed Tomography 18, no. 3 (2024): 223–232, 10.1016/j.jcct.2024.02.007. 38467535

[20] G. J. Hollands , J. A. Usher‐Smith , M. Hankins , F. Alexander , N. Clarke., and S. J. Griffin , “Visualising Health Risks With Medical Imaging for Changing Recipients' Health Behaviours and Risk Factors: Systematic Review With Meta‐Analysis,” PLoS One 19, no. 3 (2022): e1003920, 10.1371/journal.pmed.1003920. PMC8893626 35239659

[21] G. J. Hollands , D. P. French , S. J. Griffin , et al., “The Impact of Communicating Genetic Risks of Disease on Risk‐Reducing Health Behaviour: Systematic Review With Meta‐Analysis,” BMJ 352 (2016): i1102, 10.1136/bmj.i1102. 26979548 PMC4793156

[22] M. J. Frieser , S. Wilson , and S. Vrieze , “Behavioral Impact of Return of Genetic Test Results for Complex Disease: Systematic Review and Meta‐Analysis,” Health Psychology 37, no. 12 (2018): 1134–1144, 10.1037/hea0000683. 30307272 PMC6263735

[23] A. Bandura , “Self‐Efficacy: Toward a Unifying Theory of Behavioral Change,” Advances in Behaviour Research and Therapy 1, no. 4 (1978): 139–161, 10.1016/0146-6402(78)90002-4.

[24] A. Bandura , Social Foundations of Thought and Action: A Social Cognitive Theory. Prentice‐Hall, Inc, 1986, 617–xiii.

[25] A. Bandura , Self‐Efficacy: The Exercise of Control. W H Freeman/Times Books/Henry Holt & Co, 1997, 604–ix.

[26] J. Leipsic , S. Abbara , S. Achenbach , et al., “SCCT Guidelines for the Interpretation and Reporting of Coronary CT Angiography: A Report of the Society of Cardiovascular Computed Tomography Guidelines Committee,” Journal of Cardiovascular Computed Tomography 8, no. 5 (2014): 342–358, 10.1016/j.jcct.2014.07.003. 25301040

[27] A. V. Khera , C. A. Emdin , I. Drake , et al., “Genetic Risk, Adherence to a Healthy Lifestyle, and Coronary Disease,” New England Journal of Medicine 375, no. 24 (2016): 2349–2358, 10.1056/nejmoa1605086. 27959714 PMC5338864

[28] International Diabetes Federation . Diabetes Management, accessed 12 October, 2024, https://idf.org/about-diabetes/diabetes-management/healthy-nutrition/.

[29] M. C. Williams , J. Kwiecinski , M. Doris , et al., “Low‐Attenuation Noncalcified Plaque on Coronary Computed Tomography Angiography Predicts Myocardial Infarction,” Circulation 141, no. 18 (2020): 1452–1462, 10.1161/CIRCULATIONAHA.119.044720. 32174130 PMC7195857

[30] Diabetes Australia . Diabetes & Daily Life, accessed 12 October 2024, https://www.diabetesaustralia.com.au/living-with-diabetes/daily-life/.

[31] Heart Foundation . Keep Your Heart Healthy, accessed 12 October 2024, https://www.heartfoundation.org.au/healthy-living/keeping-your-heart-healthy.

[32] S. Rollnick , W. R. Miller , C. C. Butler , and M. S. Aloia , “Motivational Interviewing in Health Care: Helping Patients Change Behavior,” COPD: Journal of Chronic Obstructive Pulmonary Disease 5, no. 3 (2008): 203, 10.1080/15412550802093108.

[33] U. Näslund , N. Ng , A. Lundgren , et al., “Visualization of Asymptomatic Atherosclerotic Disease for Optimum Cardiovascular Prevention (VIPVIZA): a Pragmatic, Open‐Label, Randomised Controlled Trial,” The Lancet 393, no. 10167 (2019): 133–142, 10.1016/S0140-6736(18)32818-6. 30522919

[34] T. M. Marteau , D. P. French , S. J. Griffin , et al., “Effects of Communicating DNA‐Based Disease Risk Estimates on Risk‐Reducing Behaviours,” Cochrane Database of Systematic Reviews 2010, no. 10 (2010): CD007275, 10.1002/14651858.CD007275.pub2. 20927756 PMC12853416

[35] R. Chen , V. Pearson , O. Suebkinorn , et al., “Impact of Genetic Risk Information for Cardiovascular Disease on Behavioural, Psychological Responses, and Risk Factor Modification: A Systematic Review,” European Journal of Preventive Cardiology 33, no. 3 (2024): 327–337, 10.1093/eurjpc/zwae362. 39489494

[36] C. K. Wallingford , H. Kovilpillai , C. Jacobs , et al., “Models of Communication for Polygenic Scores and Associated Psychosocial and Behavioral Effects on Recipients: A Systematic Review,” Genetics in Medicine 25, no. 1 (2023): 1–11, 10.1016/j.gim.2022.09.008. 36322150

[37] R. Garcia‐Retamero and E. T. Cokely , “Designing Visual Aids That Promote Risk Literacy: A Systematic Review of Health Research and Evidence‐Based Design Heuristics,” Human Factors: The Journal of the Human Factors and Ergonomics Society 59, no. 4 (2017): 582–627, 10.1177/0018720817690634. 28192674

