# Supplementary material for: Evaluation of Polygenic Scores and CT Imaging in Risk Factor Modification in Patients With Diabetes: Rationale and Design of the VOLTAIRE Study
Source: Clin Cardiol. 2026 Jun 1;49(6):e70363. doi: 10.1002/clc.70363 (PMC13240284; doi:10.1002/clc.70363)
Supplement: Supplementary file 1 — Supporting File [file CLC-49-e70363-s001.docx]

# Supplementary

**Table 1 Data Collection**

| **Baseline Assessment**  Individuals who are being considered for participation in the trial undergo a comprehensive baseline assessment, conducted in person, to determine their eligibility. Baseline data collected include: |
| --- |
| 1. Medical and Family History including co-morbidities, family history of cardiovascular conditions, duration of diabetes, and current medications. 2. Socio-demographic Factors including age, sex, ethnicity, education level, living arrangements, income range, occupation and whether English is their second language, country of birth and when they moved to Australia. 3. Physical Measurements:    - Blood Pressure (BP) is measured in the upper arm using a properly fitted cuff and a calibrated automated monitor after participants have rested for at least 10 minutes.    - Body Mass Index (BMI) is determined using measurements of height (taken with a stadiometer) and weight (taken using digital scales).    - Waist Circumference is measured while the participant is standing, at the midpoint between the lowest rib and the iliac crest, following WHO guidelines STEPwise approach 4. CTCA Imaging 5. Blood Sampling:  - Routine Analysis: A blood sample is obtained to assess estimated glomerular filtration rate (eGFR), lipid profiles (total cholesterol, LDL-C, HDL-C, triglycerides, lipoprotein(a) [Lp(a)]), high-sensitivity C-reactive protein (hsCRP), and glycated haemoglobin (HbA1c). If participants have had these tests conducted within the past 14 days, results can be used for screening purposes. - Genetic Analysis: A separate blood sample is obtained to calculate the PRS.  1. Health behaviours including smoking, diet type, vegetables/fruit intake, alcohol consumption, and physical activity level. 2. Self-Reported Questionnaires:    - Psychological Health: Patient Health Questionnaire & Generalised Anxiety Disorder (PHQ-9 & GAD-7), and Diabetes Distress Score (DDS-17).    - General Health: Health-related quality of life (EQ-5D-5L), Sleep Apnoea screening (STOP-BANG), Patient Activation Measure (PAM-13), Modified VOILS questionnaire, and Health Literacy Survey (HLS-EU-12).    - Personal perceived level of cardiovascular risk and personal control.    - Women’s health survey including pregnancy history, use of hormonal contraception or hormone replacement therapy, fertility treatments, menopause experience, gynaecological conditions history. |
| **Follow-Up Assessments**  Participants undergo follow-up assessments during the study period at the following intervals: |
| 1. 6-Month Follow-Up Visit: All participants attend a follow-up visit at 6 months, which can be conducted in person, or via telehealth or phone. The study team reinforces the CTCA or PRS results for participants in the intervention groups to encourage medication compliance and positive health behavioural changes. Assessments include:    - Review of medication & cardiovascular events    - Health behaviours including smoking, diet type, vegetables/fruit intake, alcohol consumption, and physical activity level.    - Questionnaires: PAM-13 and Modified VOILS questionnaire. 2. 12-Month End-of-Study (EOS): Participants return to the clinic for an in-person EOS visit at 12 months. Assessments include:    - Repeat CTCA Imaging.    - Blood Sampling: lipid profiles, HbA1c, and hsCRP.    - Review of medication & cardiovascular events.    - Health behaviours including smoking, diet type, vegetables/fruit intake, alcohol consumption, and physical activity level.    - Self-Reported Questionnaires: PHQ-9 & GAD-7, DDS-17, EQ-5D-5L, STOP-BANG, PAM-13, Modified VOILS, Personal perceived level of cardiovascular risk and personal control, and Study evaluation survey. 3. 12-Month Phone Follow-Up End-of-Study (EOS) for Observational Registry: Participants in the observational registry undergo a follow-up phone call at the end of the 12-month study period. Similar assessments to those conducted with participants in the active study groups are performed, excluding CT imaging and blood sampling 4. Focus Group Interviews: All participants are invited to participate in a focus group interview to share their experiences and perspectives on the interventions and the study. |
